# The Inhibitory Effect of Mouse Gastric DNA on Amplification of *Helicobacter pylori* Genomic DNA in Quantitative PCR

**DOI:** 10.29252/.23.4.297

**Published:** 2019-07

**Authors:** Mehdi Alikhani, Ebrahim Shafaie, Esmat Mirabzadeh Ardakani, Maryam Esmaeili, Samaneh Saberi, Mojgan Hatefi, Marjan Mohammadi

**Affiliations:** 1 HPGC Research Group, Medical Biotechnology Department, Pasteur Institute of Iran, Tehran, Iran; 2 Animal Sciences Lab, Department of Molecular Medicine, Pasteur Institute of Iran, Tehran, Iran

**Keywords:** Amplification, Inhibition, Interference, Standard curve, Quantitative PCR

## Abstract

**Background:**

Quantitation of *Helicobacter pylori* (Hp) in the gastric tissue is essential for assessment of vaccination/therapeutic regimens.

**Methods & Results:**

Here, the inhibitory effect of mouse gastric DNA (MgDNA) on amplification of Hp genomic DNA (HpDNA) was evaluated by spiking HpDNA with serial dilutions of MgDNA, which yielded concentrations of >10 ng/µl and >0.63-10 ng/µl of MgDNA, as inhibition and interference zones, respectively. Mice were then inoculated with varying doses of Hp and assessed at the inhibition-free concentration of 0.63 ng/µl. The average Hp copy numbers per microgram of gastric tissue discriminated mice having received high vs. low dose inoculums (*p* < 0.001). Secondly, Hp copy numbers were quantitated in immunized mice, which demonstrated significantly lower numbeZrs, in reference to controls (*p* = 0.006).

**Conclusion:**

Our method, bypassing the inhibition and/or interference imposed by MgDNA, was able to quantitate gastric tissue-colonizing Hp, segregating mice inoculated with low vs. high doses of Hp, as well as those immunized from controls.

## INTRODUCTION

The success rate of any *Helicobacter pylori* (Hp)-related trial vaccination and/or therapeutic regimens is ultimately determined by quantitating the actual bacterial load, colonizing the gastric tissue. Several methods have been introduced for this objective, most of which have employed quantitative PCR (qPCR) to determine Hp genome copy numbers. The *glmM* (*ureC*) gene of Hp is most suitable for this purpose, as it is fully conserved and is present in one single copy[1]. However, the inhibiting and/or interfering effect of mammalian genomic DNA poses serious problems. Loddenkötter and colleagues[2] have reported a qPCR method, quantitating as few as 30 copy numbers of *glmM*, in the presence of excess human DNA. Roussel *et al*.[3], on the other hand, evaluated the efficiency of the quantitation of Hp 16S rDNA in mouse gastric tissue, by focusing on the extraction method, and found host DNA inhibition at minimum, when mouse tissue was homogenized by glass beads, and its DNA was extracted via commercial kits. These authors found relative quantitation (Hp/mouse genome) preferable to the absolute standard curve quantitation method[3]. Having used the relative method, Molnar *et al*.[4] observed a significant correlation with urea breath test results in infected patients and revealed higher density of colonization, in gastric regions with erosions. In 2012, Belda *et al*.[5] reported that the diagnostic accuracy of qPCR in detecting infected patients varied based on the anatomic region of the stomach, ranging from “very good” (AUC = 0.83) in the corpus to “excellent” (AUC = 0.91) in the antrum. Kargar *et al*.[6] added the analysis of point mutations in the 23S rRNA gene to the quantitation of 16S rRNA, to detect not only the bacterial load but also the rate of molecular resistance against clarithromycin. Quantitation of Hp in the gastric tissue is also of critical use, when the gastric tissue is contaminated with blood (i.e. in patients with upper gastrointestinal bleeding), which distorts the accuracy of routine diagnostic tests, such as rapid urease test (RUT)[7]. Here, we have assessed the inhibiting/interfering effect of mouse gastric DNA (MgDNA) on the quantitation of the colonizing Hp copy numbers and have been able to determine an inhibition-free threshold.

## MATERIALS AND METHODS

### Cloning of glmM

The *glmM* (*ureC*) gene fragment (978 bp) of Hp was cloned into pTG19T vector (Vivantis, Malaysia) following its amplification by *High-Fidelity* DNA polymerase (TransTaq, TransGen Biotech, China), using *glmM*-specific primers (F: 5′- GAT GGC GTG AGG GGT AAA G-3′ and R: 5′-GATATG CCC GCT TTG CTC-3′), under the following conditions: 4 min at 94 ºC as initial denaturation, followed by 30 cycles of 60 s at 94 ºC for denaturation, 30 s at 57.4 ºC as annealing, 60 s at 72 ºC for extension, and final extension at 72 ºC for 10 min. The recombinant pTG19T-*glmM* vector was transformed into fresh competent *E. coli* TOP10F’ strain (Invitrogen, USA) and cultured on LB agar plates, supplemented with 100 µg/ml of ampicillin, 15 µg/ml of tetracycline, 1 mM of isopropyl B-D-1-thiogalactopyranoside (IPTG), and 40 µg/ml of X-gal. The Blue-white screening was performed to select *glmM*-positive clones. The cloning procedure was further confirmed by PCR and enzymatic (*Bam*HI) digestion.

### Generation of standard curves

The circular pTG19T*-glmM* vector was linearized by *Xba*I enzyme, and its mean concentration was determined by OD_260_ measurement, in triplicates (Epoch Microplate spectrophotometer, USA). The mass and copy number of pTG19T*-glmM* was calculated by the Applied Biosystems (ABI, USA) recommended formula[8]: Mass = (plasmid size [bp]) × (1.096 × 10^-21^). Since there was a single copy of the *glmM* gene in each plasmid template (PT) and Hp genomic DNA (HpDNA), the mass required for the preparation of serial copy number dilutions (range: 10-10^9^ copies) was calculated. HpDNA was extracted by DNA extraction kit (MN, Germany), and the required DNA for serial dilutions (10-10^8^ of HpDNA copies) was prepared, as mentioned for PT, keeping in mind that the average size of HpDNA is 1.6 Mb.

### Quantitative real-time PCR

Serial dilutions of PT and HpDNA underwent amplification by qPCR, which was performed in total reaction volumes of 10 µl, containing 5 µl of Fast SYBR® Green Master Mix (ABI, USA), 5 pM of each *glmM* primer (RT-F: 5’ ATG TTT GTG ATG CGT TTA 3’ and RT-R: 5’ AGC CTA TGG AAG TGA GAG 3’), and DNA templates (serial dilutions of PT, HpDNA, and MgDNA). qPCR was conducted by Applied Biosystems StepOne™ Real-Time PCR Systems, under the following conditions: 14 min at 95 ºC as initial denaturation, followed by 50 cycles of 15 s at 95 ºC for denaturation, and 60 s at 60 ºC for annealing and extension. Melting curve analysis was assessed for the 60-95 ºC temperature range, and the cycle threshold (Ct) was calculated by StepOne v3.2 software (ABI, USA).

### The inhibitory effect of mouse gastric DNA

The inhibiting role of MgDNA on *glmM* gene amplification was investigated via spiking 10^5^ copies of PT and HpDNA, with 14 serial dilutions (0-20 ng/µl) of MgDNA in qPCR reaction and co-amplified, as mentioned above. The inhibiting impact of MgDNA was investigated by Ct calculation and copy number quantitation, in the presence of various concentrations.

### Quantitation of Hp copy numbers in mice

Three groups of 4-6-week-old female C57BL/6 mice (five per group) were orally inoculated thrice in three consecutive days with different doses (3 × 10^4^, 3 × 10^5^, and 3 × 10^6^ CFU/mouse) with mouse-adapted Hp. Immunization was performed, as described previously[9], with minor modifications [10]. Briefly, mice were orally intubated with inoculums containing 10 µg of cholera toxin and 100 µg of recombinant Urease B subunit (UreB)[11], in 250 µl of gavage buffer (0.5% casein hydrolysate, 0.2 mol/L of sodium bicarbonate, and 0.5% glucose in PBS), thrice with two-week intervals. The sham-immunized control group received only the gavage buffer. Two weeks following the last immunization, both groups were inoculated with 3 × 10^5^ CFU of 24-hour old, liquid cultured Hp[12], and sacrificed two weeks thereafter. At sacrifice, mouse stomachs were longitudinally divided into two parts for: (1) RUT and (2) DNA extraction. RUT color development was documented at 4 h. Total DNA was extracted from snap-frozen tissue by a commercial kit (MN, Germany). Optimized concentrations were used for quantitation of the tissue- colonizing Hp by qPCR. Standard curves of PT and HpDNA were employed to calculate the Hp copy numbers per microgram of gastric tissue.

## RESULTS

### Generation of standard curves

**Fig. 1:**
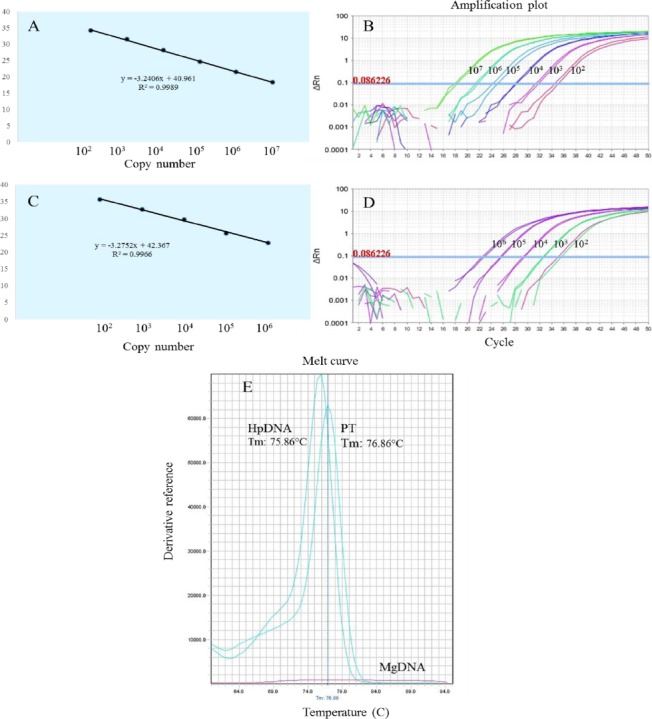
Standard curves for *glmM* plasmid template (PT) and HpDNA. (A) Linear regression of PT, (B) the amplification plot of PT serial dilutions, (C) linear regression of HpDNA, (D) the amplification of HpDNA serial dilutions, and (E) melting curve analysis for PT, HpDNA, and MgDNA.

Gene-specific amplification was successful for 10^1^-10^9^ copies of PT. Regression analysis showed a linear pattern of Ct for 10^2^-10^7^ copy numbers of PT, with a correlation coefficient (R^2^) of >0.99 (Fig. 1A and 1B). The same was applied for HpDNA gene-specific quantitation, which amplified 10^1^-10^7^ copy numbers of HpDNA and demonstrated a linear pattern for 10^2^-10^6^ copies of HpDNA (R^2^ > 0.99, Figs. 1C and 1D). Considering the fact that SYBR Green binds to double- stranded DNA, SYBR Green-based qPCR is unable to discriminate between specific and non-specific PCR products. However, our melting curve analysis confirmed the specificity of the amplified *glmM* gene product, with melting temperatures of 76.86 ºC for PT and 75.86 ºC for HpDNA, whereas no melting temperature was observed for MgDNA (Fig. 1E).

### The inhibitory effect of MgDNA on the amplification of PT and HpDNA

**Fig. 2:**
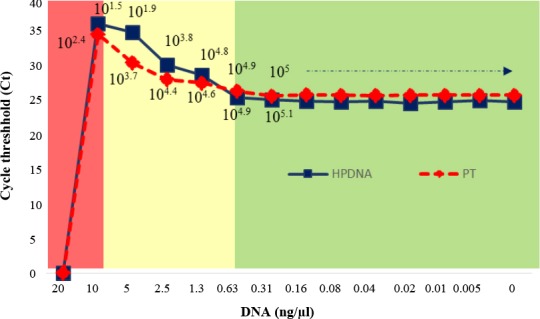
The inhibitory effect of MgDNA on amplification of PT and HpDNA cycle threshold (Ct) and the resulting copy numbers of PT (dotted line) and HpDNA (solid line), in the presence of 0-20 ng/µl of MgDNA; the inhibition (>10 ng/µl), interference (>0.63-10 ng/µl), and inhibition-free (≤0.63 ng/µl) zones are denoted.

In order to assess the inhibitory effect of MgDNA on amplification of PT and HpDNA, 10^5^ copies of each were spiked with 14 serial dilutions of MgDNA (0-20 ng/µl). This analysis revealed (1) inhibition (>10 ng/µl), (2) interference (>0.63-10 ng/µl), and (3) inhibition-free (≤0.63 ng/µl) zones, which coincided for both PT and HpDNA amplification (Fig. 2).

### Quantitation of Hp copy numbers in mice

**Fig. 3:**
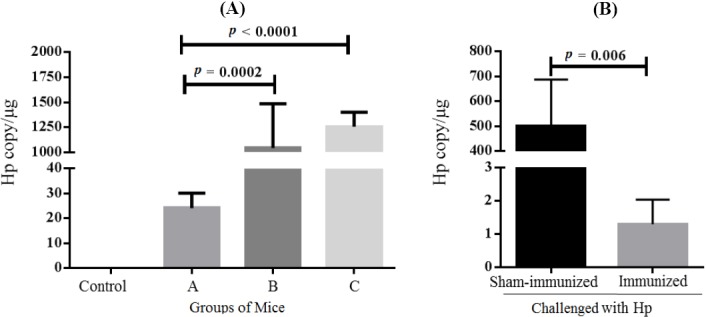
Quantitation of Hp copy numbers in the mouse gastric tissue. (A) Groups of mice inoculated with 3 × 10^4^ (Group A), 3 × 10^5^ (Group B), and 3 × 10^6^ (Group C) CFU of Hp, in comparison with uninfected controls. (B) Groups of immunized vs. sham-immunized control mice, challenged with Hp.

The sensitivity of our method was evaluated by the quantitation of the colonizing Hp in groups of mice, inoculated with varying doses of Hp: (Group A) 3 × 10^4^, (Group B) 3 × 10^5^, and (Group C) 3 × 10^6^ CFU. According to RUT results, every tissue sample underwent color changes with varying intensities over time, ranging from lowest (Group A) to highest (Group C). Keeping in mind the highest inhibition-free concentration of MgDNA, we quantitated Hp copy number in 0.63 ng/µl of mouse gastric DNA. Accordingly, the average Hp copy numbers per microgram of gastric tissue for each group of mice was ascertained as: (Group A) 24 ± 6, (Group B) 1042 ± 442, and (Group C) 1255 ± 145, which discriminated mice in group A from those in groups B (*p* = 0.0002) and C (*p* < 0.0001, Fig. 3A). Thereafter, we employed this concentration of MgDNA (0.63 µg/ml) to quantitate Hp copy numbers in a vaccine-mouse model. For this purpose, immunized (UreB + cholera toxin) and sham-immunized mice were challenged with Hp and sacrificed two weeks later. RUT test results exhibited a highly intense color development for the sham-immunized mice, whereas the immunized mice revealed no color change. Once again, 0.63 ng/µl of MgDNA underwent Hp copy number quantitation per microgram of gastric tissue and yielded significantly lower copy numbers for immunized mice (1.2 ± 0.7), as compared to the controls (499 ± 187, *p* = 0.006, Fig. 3B).

## DISCUSSION

Quantitation of Hp in gastric tissue plays an essential role in the evaluation of any vaccination and/or therapeutic regimens. There are various molecular methods for Hp quantitation in biological samples, such as competitive PCR[13], noncompetitive PCR[14], and fluorescence-based PCR technology (real-time PCR)[5,7]. The major cause of PCR failure is the presence of PCR inhibitors, which can lead to false-negative results[15]. In this study, we have extensively evaluated the inhibitory effect of MgDNA on HpDNA amplification. It is known that the amplification of DNA from colonized Hp in gastric tissue is inhibited by gastric tissue DNA[3,16]. Inhibition in real-time PCR can result from PCR inhibitors within extraction reagents[3] and from excess of non-target DNA concentration[3,15,17,18], which may affect primer annealing efficiency and interference with SYBR Green I to dsDNA binding[15]. The most common method of avoiding the inhibitory effect is optimization of the template content to an inhibition-free concentration[3,15].

Roussel *et al*.[3] performed a comprehensive study evaluating different extraction methods and found that the lowest inhibition is observed in samples homogenized by vortexing and purified by commercial column-based kits. They also assessed the inhibitory effect of non-target DNA by amplification of bacterial 16S gene in the presence of 20 ng/µl of MgDNA, which resulted in 30% inhibition. Moreover, they prepared 1:10 and 1:100 dilutions of MgDNA and observed more than 90% detection of bacterial 16S plasmid DNA in the diluted samples. However, they did not determine the exact concentration of the diluted samples. In this study, we were able to identify three zones, where the amplification of HpDNA was faced with (1) full inhibition (>10 ng/µl), (2) partial inhibition or interference (0.63-10 ng/µl), and (3) no inhibition with ~100% detection rate (≤0.63 ng/µl). According to this accurate assessment, at estimated concentration of 0.63 ng/µl of mouse gastric tissue DNA, HpDNA could be fully quantitated by qPCR. This finding is of particular importance when the ratio of HpDNA to MgDNA is at minimal levels[3,19,20].

Quantitation of Hp copy number in the stomachs of treated mice is an important approach for the evaluation of vaccination/treatment efficacy[16]. Our developed method could successfully differentiate mice infected with different doses of Hp inoculums. Furthermore, it was applied for Hp quantitation in immunized vs. sham-immunized mice and could show significant differences between the two groups. Our method could amplify 10^1^-10^7^ and 10^1^-10^9^ copies of HpDNA and PT, respectively. Nevertheless, the Ct of 10 copies did not linearly follow the previous dilutions. Therefore, the quantitation of low Hp copy numbers in the stomachs of colonized mice requires further optimization.

In conclusion, we have hereby developed a method for Hp copy number quantitation, which can bypass the interference and/or inhibition imposed by MgDNA and is able to quantitate the colonizing Hp copy numbers in the gastric tissue, segregating mice inoculated with low vs. high doses of Hp, as well as those immunized vs. controls.
